# Supplementary material for: Cryo-EM structures of KdpFABC suggest a K+ transport mechanism via two inter-subunit half-channels
Source: Nat Commun. 2018 Nov 26;9:4971. doi: 10.1038/s41467-018-07319-2 (PMC6255902; doi:10.1038/s41467-018-07319-2)
Supplement: Supplementary file 1 — Supplementary Information [file 41467_2018_7319_MOESM1_ESM.pdf]

Supplementary Information for:

Cryo-EM structures of KdpFABC suggest a K^+ transport mechanism via two inter-subunit half-channels

C. Stock, L. Hielkema, I. Tascón et al.

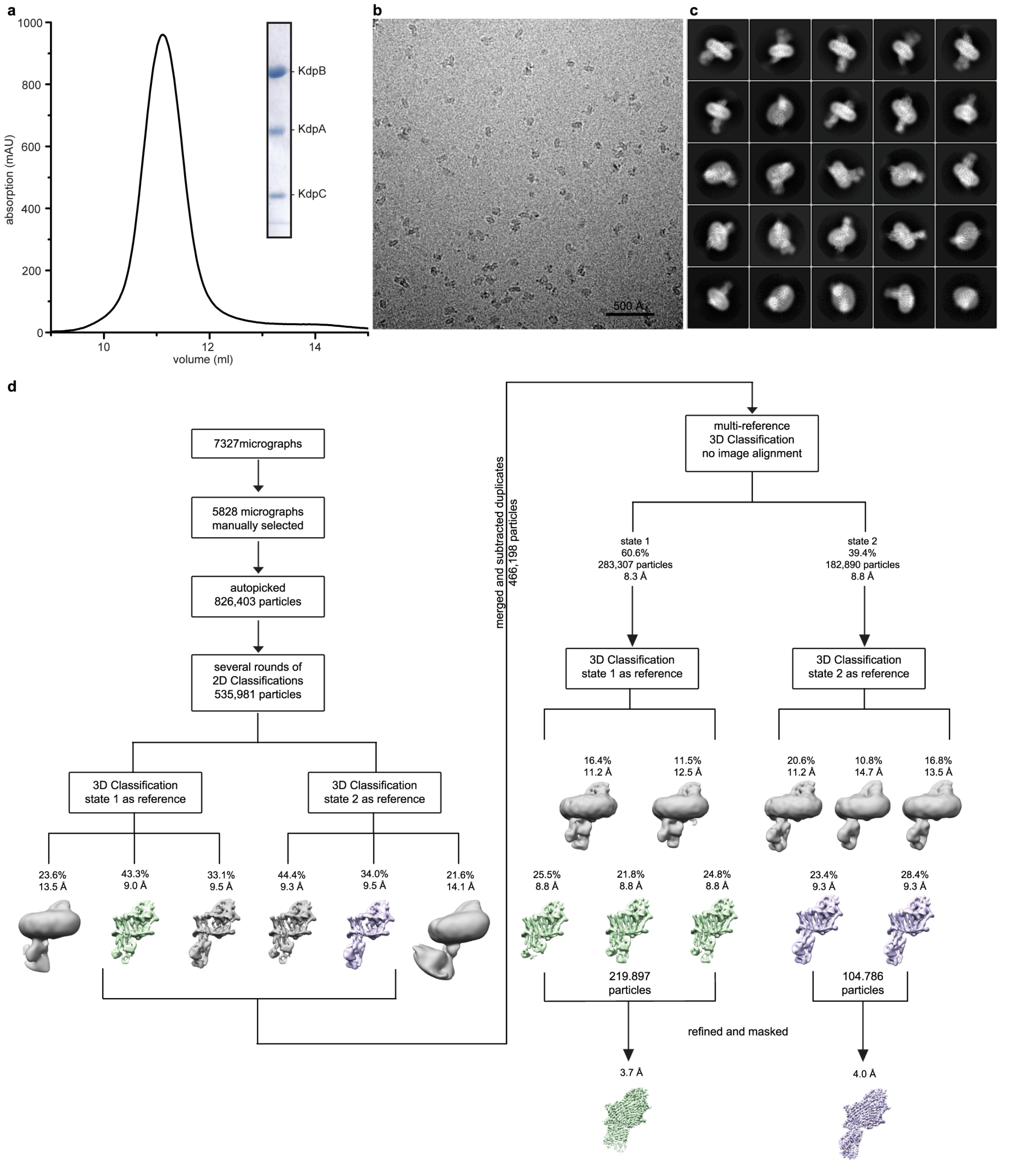

Supplementary Figure 1 Cryo-EM analysis of the KdpFABC complex. a, Size exclusion chromatography of DDM-solubilized KdpFABC used for cryo-EM studies. The inset shows a SDS-PAGE of purified KdpFABC. KdpF is not visible due to its size of 3 kDa. **b,** Representative cryo-EM image and **c,** 2D-class averages of vitrified KdpFABC in the presence of KCl, AMPPCP and AlF_4^-. **d,** Image processing work flow as described in material and methods.

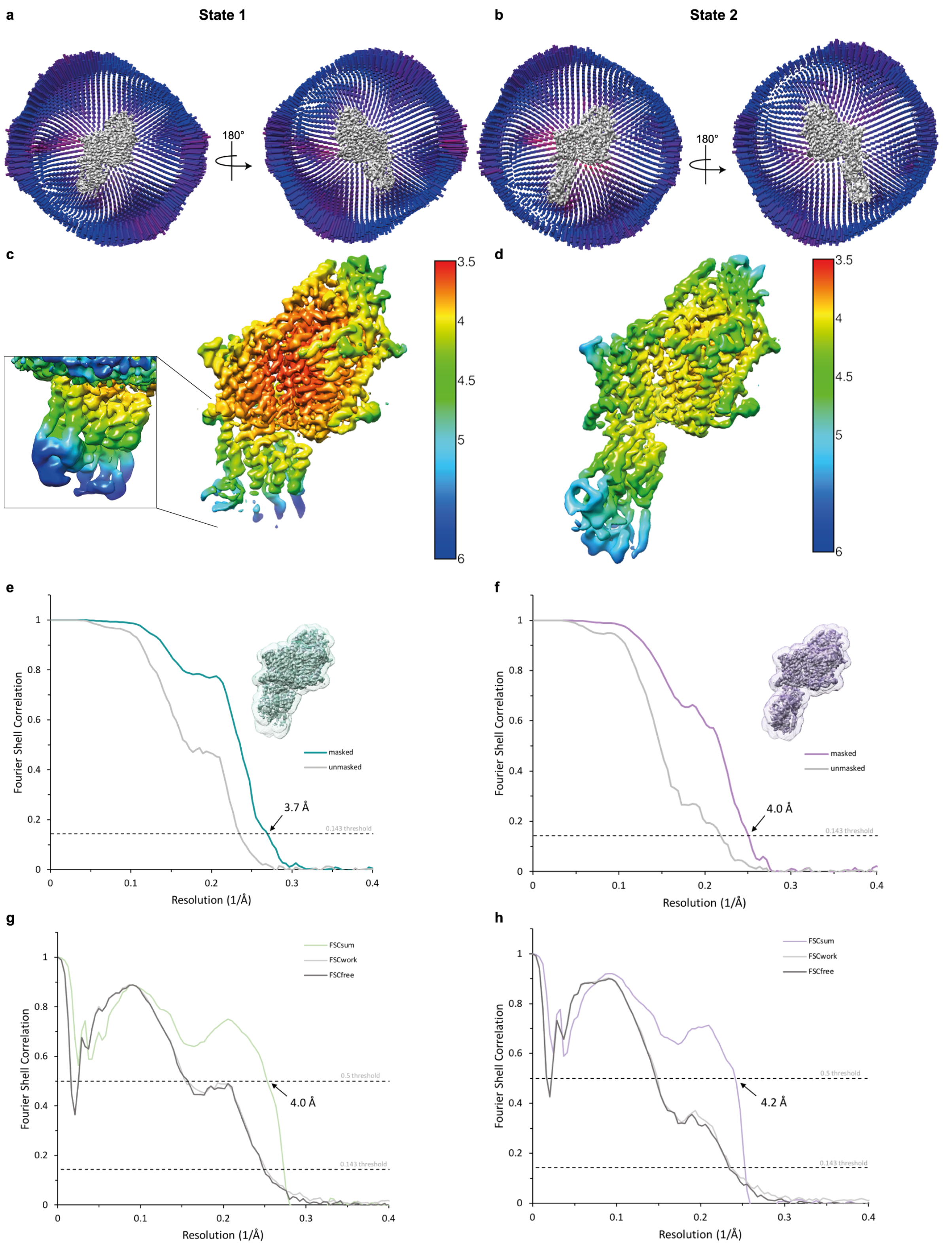

Supplementary Figure 2 Cryo-EM reconstruction. Angular distribution plot of particles included in the final unsymmetrized 3D reconstruction for KdpFABC **a,** state 1 and **b,** state 2. The number of particles with the respective orientations is represented by length and color of the cylinders (long and red: high number of particles; short and blue: low number of

particles). Final reconstruction map colored by local resolution as estimated by Relion[1,2] for **c,** state 1 and **d,** state 2. Inset in (c) shows N, P and A domains at higher contour level. FSC plot of the final refined unmasked (grey) and masked (colored) map for state 1 (**e,** green) and state 2 (**f,** violet). The resolution at which the curves drop below the 0.143 threshold is indicated. A thumbnail of the mask used for FSC calculation overlaid on the atomic model is shown in the upper right corner. **g and h,** FSC validation curves. FSC curves calculated between (g) the refined models for state 1 and (h) state 2 versus the corresponding cryo-EM maps. Green or violet: FSC curves for the refined model compared to the full masked dataset (FSCsum); light grey, FSC curve for the refined model compared to the unmasked half-map 1 (FSCwork, used during validation refinement); dark grey, refined model compared to the unmasked half-map 2 (FSCfree, not used during validation refinement). Dashed lines, FSC threshold used for FSCsum of 0.5 and for FSCfree/work of 0.143.

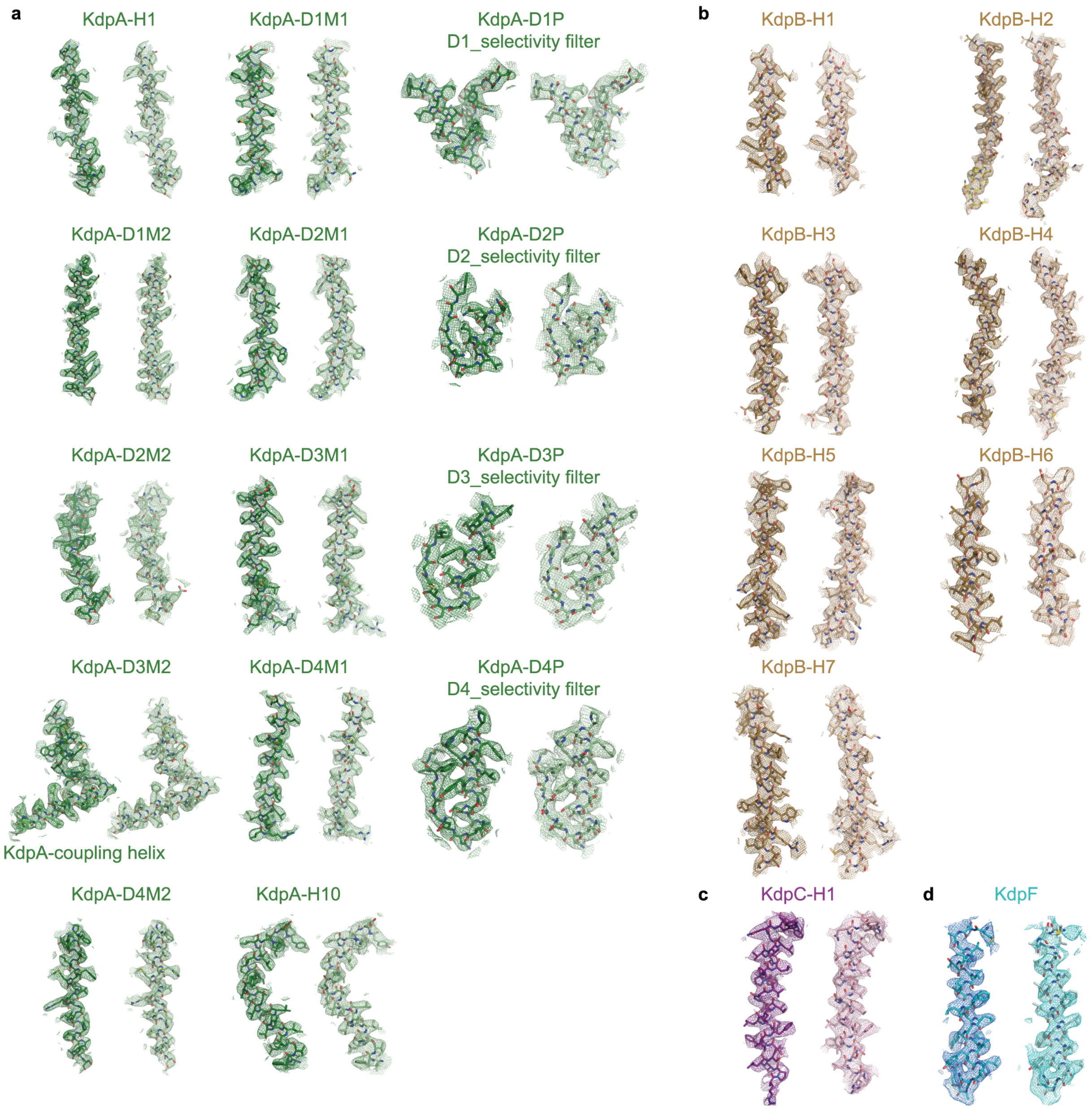

Supplementary Figure 3 Cryo-EM densities of the membrane-inserted moieties of KdpFABC. a, TM helices 1-10 and selectivity filter pore loops of KdpA, **b,** TM helices 1-7 of KdpB, **c,** TM helix of KdpC and **d,** KdpF in state 1 (left) and state 2 (right) are fitted to the corresponding maps sharpened with a b-factor of -205 Å^2 and -195 Å^2, respectively, and plotted at 7σ.

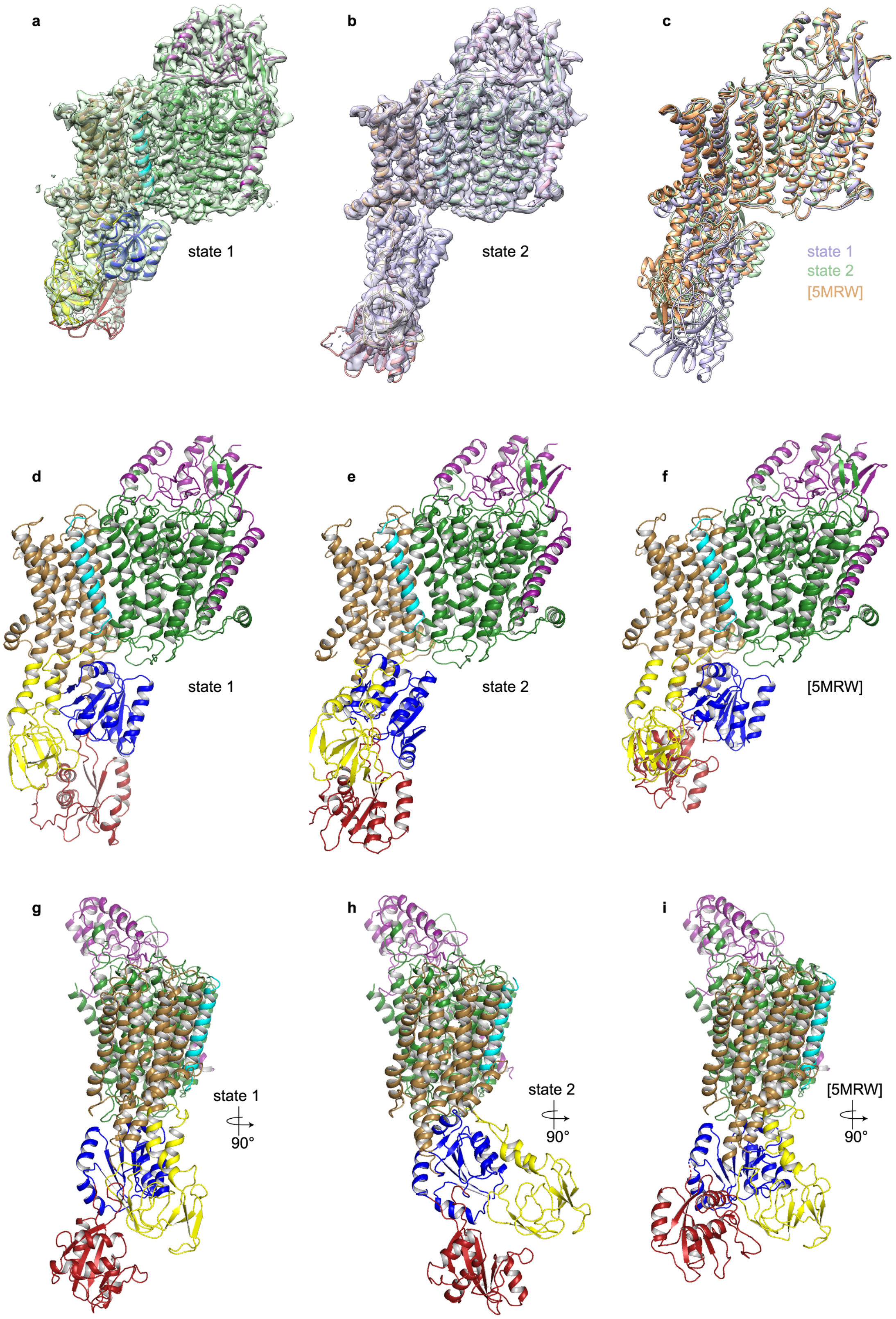

Supplementary Figure 4 Conformational states of KdpFABC. Models of state 1 (**a, d and g**), state 2 (**b, e and h**) and the crystallographic structure [5MRW] (**f and i**). Superposition of the models on the respective cryo-EM maps (**a and b**) or on the crystallographic structure

[5MRW, orange] (**c**). Structures are shown from the front (**a-f**) or from the side rotated by 90 degrees (**g-i**). Cryo-EM density map of state 1 is shown in green, map of state 2 is shown in violet. All structures were superimposed on the transmembrane domains and shown in the respective color code as in Figure 1, expect for the superposition in (c) where state 1 is shown in green, state 2 in violet and the crystallographic structure is depicted in orange.

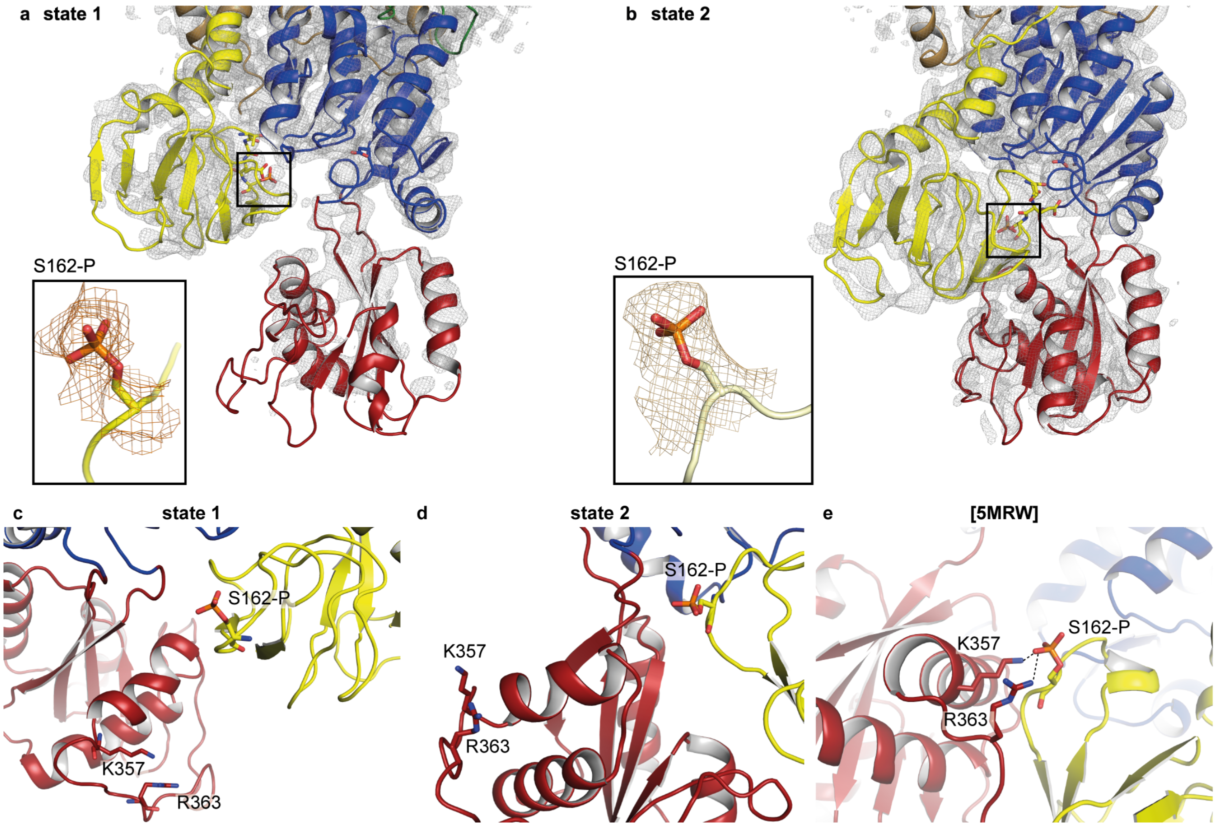

Supplementary Figure 5 Cytoplasmic domains of KdpFABC and Ser162-P. N, P and A domains of KdpB shown with cryo-EM density map (grey) in state 1 (**a,** b-factor of -154 Å^2 at 5.5σ) and state 2 (**b,** b-factor of -147 Å^2 at 6.5σ). Shown as sticks are the TGES motif, including phosphorylated Ser162, and catalytic phosphorylation site Asp307. Presented views are identical to Figure 1c and d. Insets show an enlarged view of Ser162-P with cryo-EM density in a rotated orientation. **c,** Positioning of Ser162-P, Lys357, Arg363 in state 1. **d,** Position of Ser162-P, Lys357 and Arg363 in state 2. **e,** Salt bridges observed between Ser162-P, Lys357 and Arg363 in the crystal structure [5MRW].

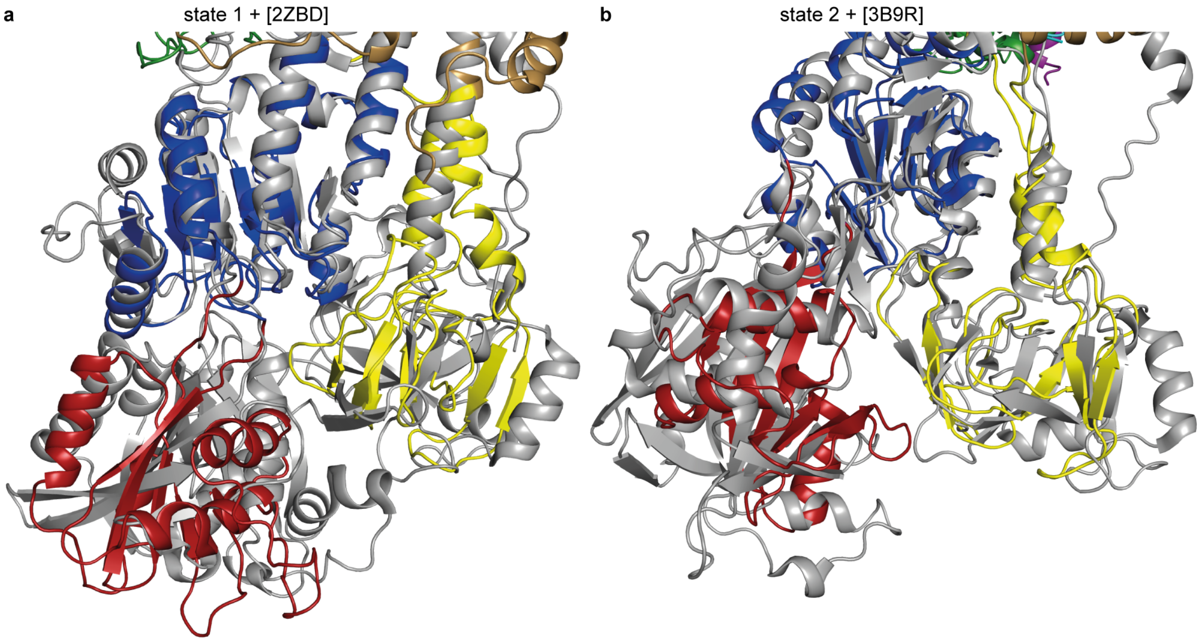

Supplementary Figure 6 Superposition of KdpB with structures of SERCA. a, Superposition of N, P and A domains of KdpB of state 1 and the SERCA structure [2ZBD] stabilized in a Ca^{2+}-E1-ADP-AlF_4^- conformation. **b,** Superposition of N, P and A domains of KdpB of state 2 with the SERCA structure [3B9R] inhibited in an E2-AlF_4^--AMPPCP conformation. In state 1 and 2, KdpB was shown to align best with SERCA structures [2ZBD] and [3B9R], respectively, when comparing them with 12 SERCA structures from *Oryctolagus cuniculus* (Supplementary Table 2).

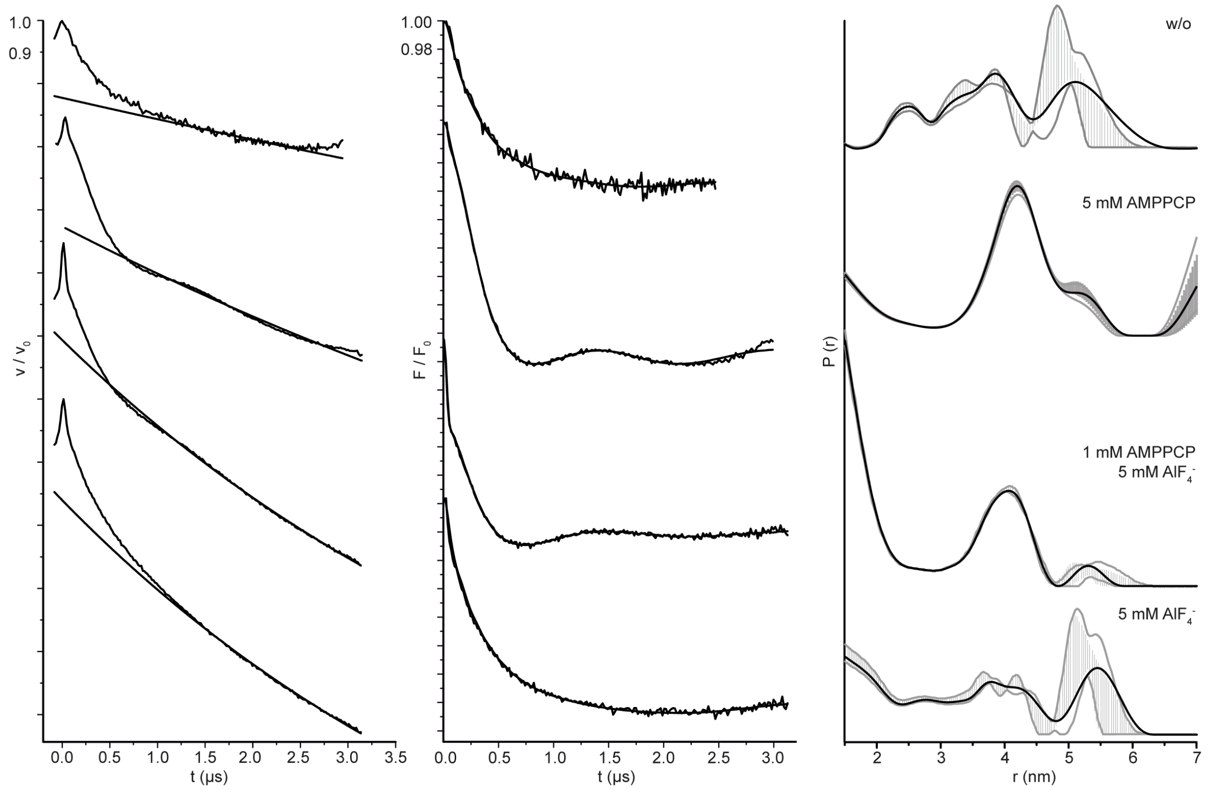

Supplementary Figure 7 Pulsed EPR measurements of variant KdpFAB$_{G150CR1/A407CR1}$C in the absence and presence of different inhibitors. Panel 1: experimental raw data V(t). Panel 2: dipolar evolution function F(t). Panel 3: interspin distance distribution P(r) (black lines) obtained by Tikhonov regularization. Grey areas indicate the full variation of possible distance distributions. The lower and upper error estimates (grey lines) represent the respective mean value minus and plus two times its standard deviation, respectively. The larger the deviations, the less reliable are the predicted distances.

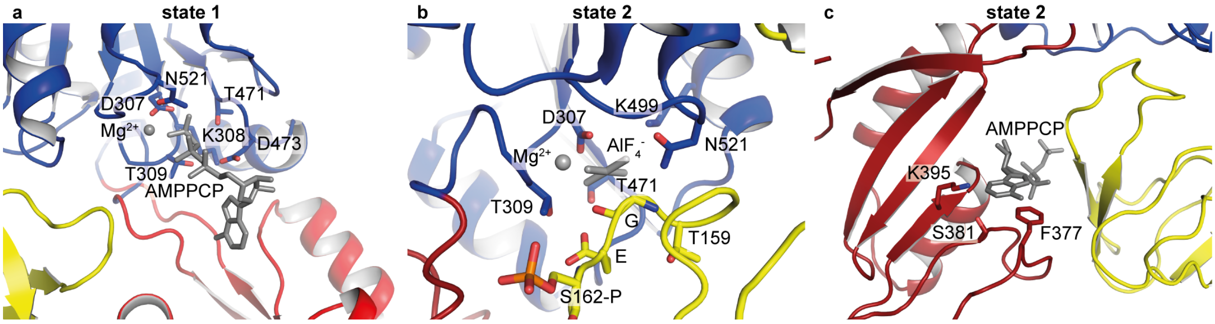

Supplementary Figure 8 Close up views of state 1 and 2 highlighting docked ligands. a, Possible coordination of AMPPCP-Mg^{2+} in state 1 by residues Asp307, Lys308, Thr309, Thr471, Asp473, and Asn521. AMPPCP-Mg^{2+} was docked based on the SERCA structure [1T5S]. **b,** Docked Mg^{2+} and AlF_4^- coordinated by Asp307, Thr309, Thr471, Lys499 and Asn521 of the P domain and Glu161 of the A domain in state 2. **c,** Potential modulatory AMPPCP binding pocket near Phe377, Lys395 and Ser381 in state 2. Mg^{2+} and AlF_4^- as well as AMPPCP in b and c, respectively, docked based on comparison with the SERCA structure [3B9R].

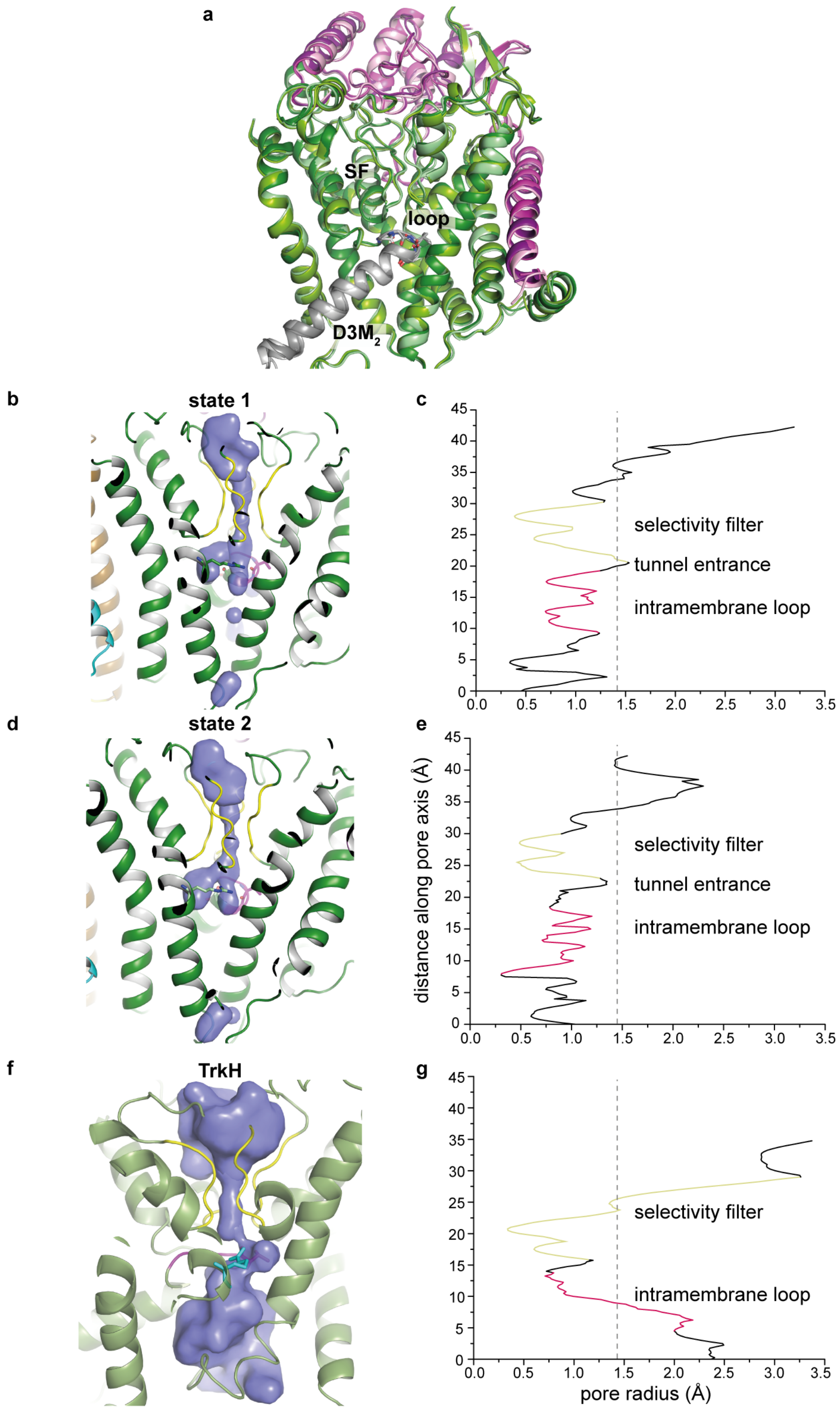

Supplementary Figure 9 Rigidity of KdpA's hypothetical pore. a, Rigid structure of KdpA's hypothetical pore with selectivity filter (SF), intramembrane loop (with residues Gly374, Gly375, Val376 and Gly377 shown as sticks), coupling helix $D3M_2$, and KdpC. Superposition of state 1, state 2 and crystal structure [5MRW] (KdpA in bright, pale and light green, KdpC in purple, mauve and rose). No structural changes are observed. **b, d, f,**

Visualization of calculated pore diameters for KdpA in state 1, state 2 and for TrkH [3PJZ][3]. **c, e, g,** Correlating pore radii through the presumed translocation pores of KdpA and TrkH, respectively. Pore densities were calculated with HOLLOW[4] and pore radii determined by HOLE[5]. Highlighted selectivity filter in yellow, intramembrane loop in purple, and Arg493 (KdpFABC) or Arg468 (TrkH) in turquois. Pore densities depicted in purple, transmembrane helices of KdpA/TrkH in green. Dotted grey line marks the radius of a dehydrated K^+.

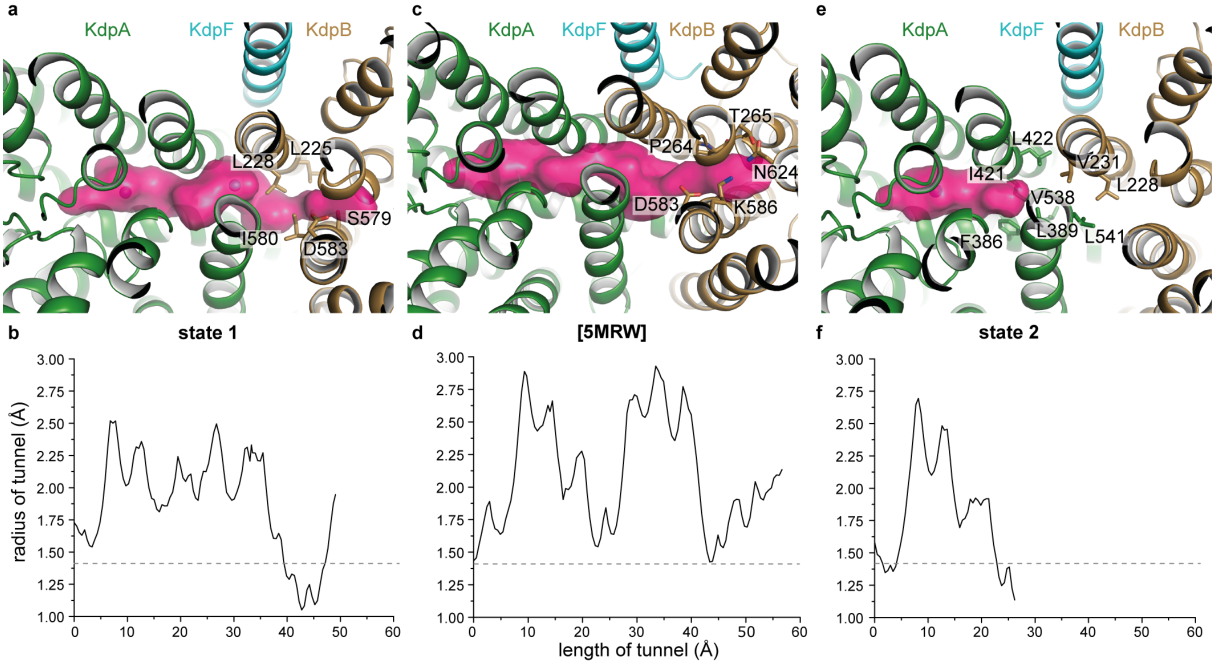

Supplementary Figure 10 Comparison of inter-subunit tunnels connecting KdpA to KdpB. a, Inter-subunit tunnel observed in state 1 and **b,** its tunnel radii. The tunnel radius is restricted below 1.5 Å within KdpB hindering the access of K^+ to the canonical binding site (residues that form the restriction site are depicted as sticks and labeled). **c,** Inter-subunit tunnel ranging from the entry site in KdpA to the canonical binding site in KdpB (residues of binding site are depicted as sticks and labeled) as observed in the crystallographic structure [5MRW] and **d,** its tunnel radii. **e,** Inter-subunit tunnel as observed in state 2 and **f,** its tunnel radii. Residues at the KdpA/KdpB interface completely close the tunnel, blocking any K^+ passage (blocking residues are depicted as sticks and labeled). Tunnel densities were calculated with HOLLOW[4], and tunnel radii determined by CAVER[6]. Dotted grey line marks the radius of dehydrated K^+.

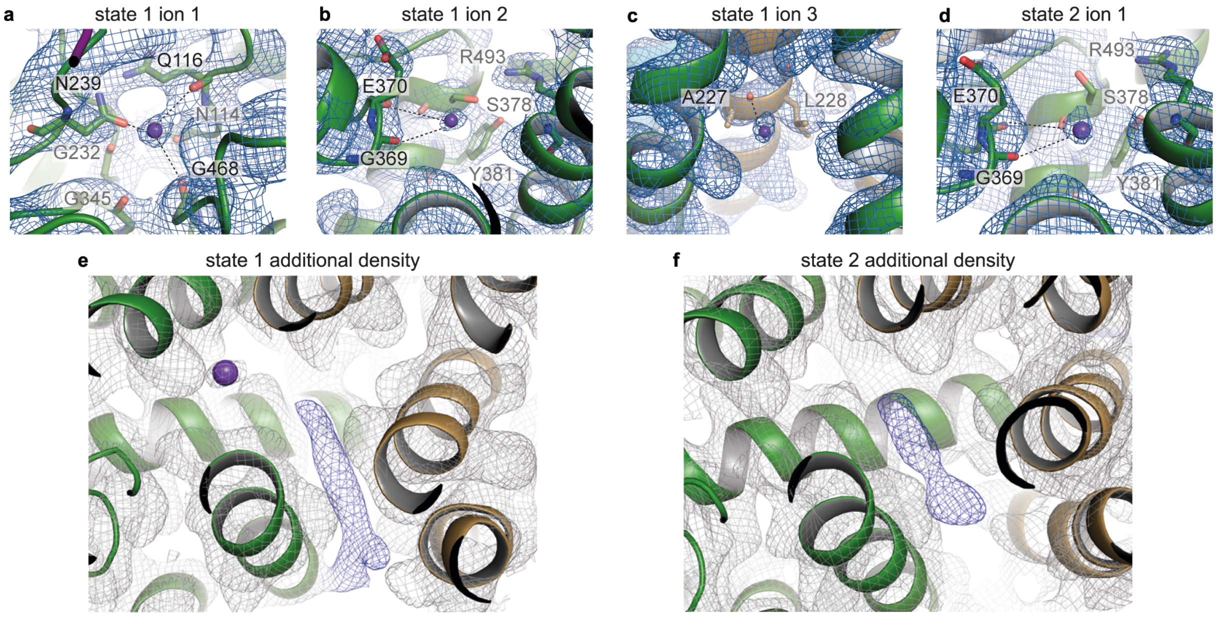

Supplementary Figure 11 Additional ligands shown by cryo-EM densities. a-d, Identical to Figure 2 b-d and g. The cryo-EM density for the potassium ions and the protein is shown at identical b-factors and σ. **e and f,** Additional density most likely corresponding to co-purified lipids at the interface between KdpA and KdpB in state 1 (**e**, b-factor of -205 $Å^2$ at 5σ) and state 2 (**f**, b-factor of -195 $Å^2$ at 6σ), depicted as blue mesh while surrounding densities are grey.

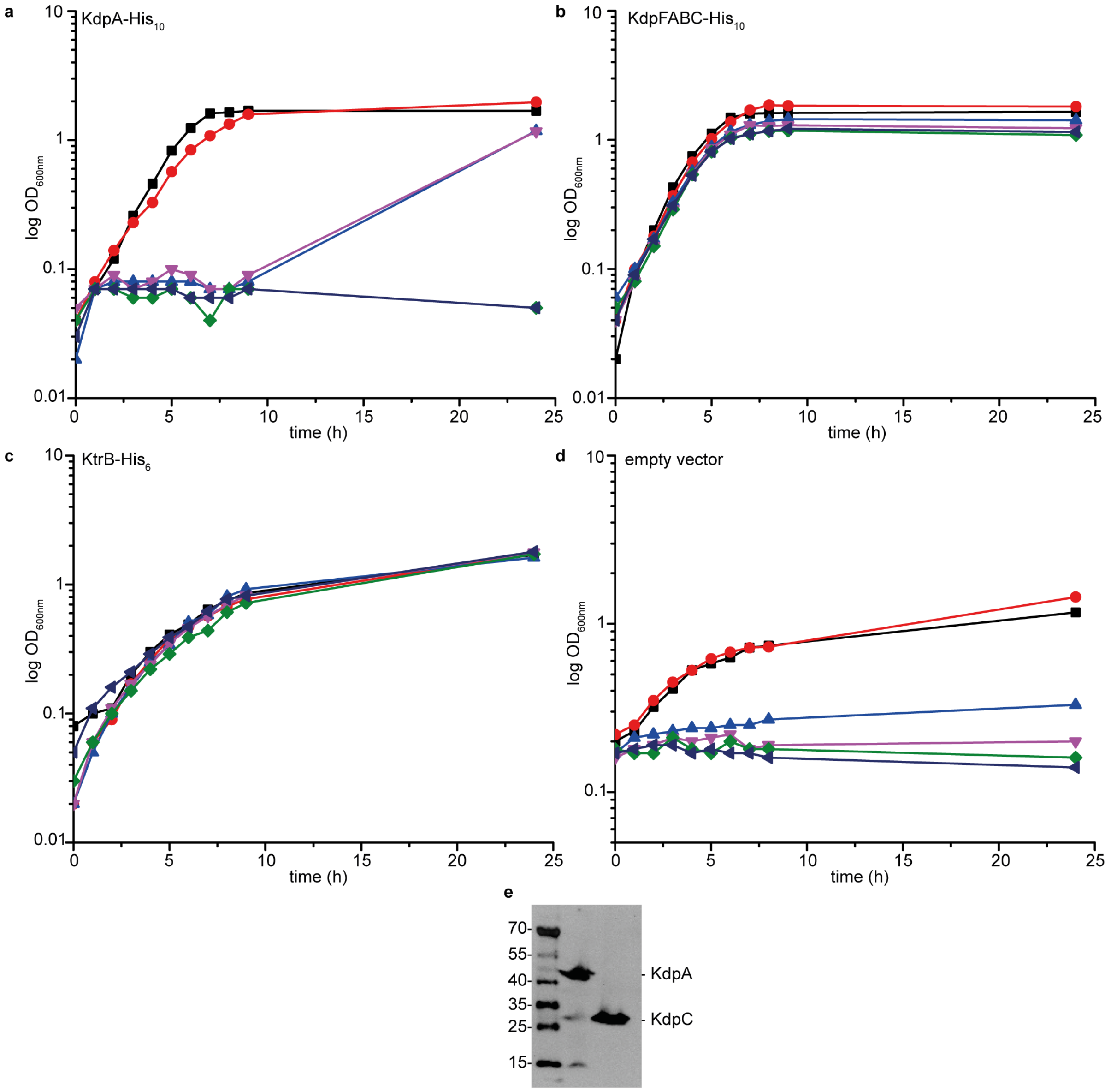

Supplementary Figure 12 Growth complementation assay for the determination of potential K^+ uptake via KdpA a, LB2003/p7XC3H-KdpA encoding C-terminally His_{10}-tagged KdpA. **b,** LB2003/p7XC3H-KdpFABC encoding C-terminally His_{10}-tagged KdpFABC. **c,** LB2003/pEL903 encoding C-terminally His_6-tagged KtrB. **d,** LB2003/pBAD18 representing an empty vector control. Minimal media with K^+ concentrations of 115 mM (black square), 30 mM (red circle), 10 mM (blue triangle), 7 mM (pink triangle), 3 mM (green diamond), 1 mM (dark blue triangle) was used and growth curves were recorded. While the production of KdpFABC and the channel-like KtrB subunit were sufficient to complement the strain's lack of K^+ uptake systems and allowed cell growth at low potassium concentrations, KdpA-alone expressing cells did not grow below 10 mM K^+. **e,** Western Blot analysis of protein production. Applied cells were grown in minimal media containing 30 mM K^+. Production of KdpA in LB2003/p7XC3H-KdpA and KdpFABC in LB2003/p7XC3H-KdpFABC was confirmed.

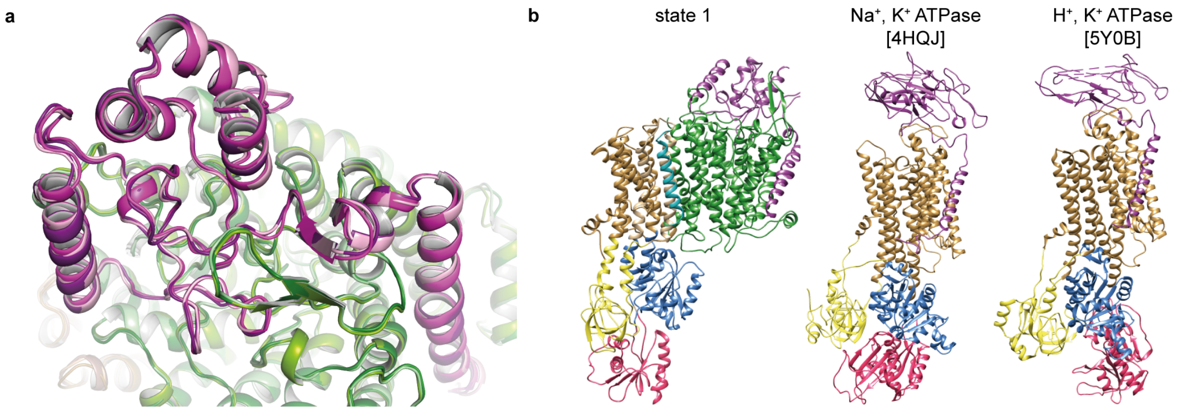

Supplementary Figure 13 Structural comparison of KdpC and the β subunits of type IIC P-type ATPases. a, Superposition of KdpC in state 1, state 2 and the crystallographic structure [5MRW] depicted in different shades of purple. No obvious structural changes are visible. **b,** Comparison of the overall structures of KdpFABC (state 1), Na^+,K^+-ATPase [4HQJ][7] and gastric H^+,K^+-ATPase [5Y0B][8]. In particular the resemblance in local proximity of KdpC and β subunits with respect to the entry site of the ions is striking. KdpC and β subunits depicted in purple.

Supplementary Table 1 Cryo-EM data collection, refinement and validation statistics.

	KdpFABC State 1 (EMDB-0257) (PDB 6HRA)	KdpFABC State 2 (EMDB-0258) (PDB 6HRB)
**Data collection and processing**		
Magnification	49,407	49,407
Voltage (kV)	200	200
Electron exposure (e–/Å^2)	52	52
Defocus range (μm)	-0.4 to -3	-0.4 to -3
Pixel size (Å)	1.012	1.012
Symmetry imposed	C1	C1
Initial particle images (no.)	535,981	535,981
Final particle images (no.)	219,897	104,786
Map resolution (Å)	3.7	4.0 (0.143)
FSC threshold	0.143	0.143
Map resolution range (Å)	3.5-6	3.8-6
**Refinement**		
Initial model used (PDB code)	5RMW	5RMW
Model resolution (Å)	4.0	4.2
FSC threshold	0.5	0.5
Model resolution range (Å)	80-3.7	80-4.0
Map sharpening *B* factor (Å^2)	-154	-147
Model composition		
Non-hydrogen atoms	10818	10787
Protein residues	1450	1447
Ligands	3	1
B factors (Å^2)		
Protein	116.7	128.4
Ligand	39.3	31.4
R.m.s. deviations		
Bond lengths (Å)	0.007	0.008
Bond angles (°)	0.899	0.950
Validation		
MolProbity score	1.83	1.83
Clashscore	6.64	6.75
Poor rotamers (%)	0.18	0.26
Ramachandran plot		
Favored (%)	92.6	92.8
Allowed (%)	7.4	7.2
Disallowed (%)	0	0

Supplementary Table 2 Comparison between N, P and A domains of KdpB in state 1 and 2 and SERCA in different conformations.

PDBs	inhibitors	state	RMSD state 1	RMSD state2
4H1W [9]	K^+, Mg^{2+}, AMPPCP	E1	3.91	4.37
1T5S [10]	K^+, Mg^{2+}, AMPPCP, Ca^{2+}	(Ca)E1-ATP	3.29	3.92
1T5T [10]	K^+, Mg^{2+}, ADP, AlF_4^-, Ca^{2+}	(Ca)-E1P-ADP	**2.95**	4.14
2ZBD [11]	Mg^{2+}, ADP, AlF_4^-, Ca^{2+}	(Ca)-E1P-ADP	3.04	4.29
3BA6 [12]	K^+, Ca^{2+}, AMP Phosporamidate	(Ca)-E1P:ADP	3.33	3.25
3B9B [12]	Na^+, Mg^{2+}, BeF_3^-	E2P	4.07	3.20
2ZBF [13]	Tg, Mg^{2+}, BeF_3^-	E2P	4.07	3.08
3B9R [12]	K^+, Mg^{2+}, AMPPCP, AlF_4^-	E2-P	3.31	2.13
1XP5 [14]	K^+, Mg^{2+}, Tg, AlF_4^-	E2-P	4.43	**2.07**
1WPG [15]	Tg, Na^+, MgF_4^{2-}, ADP, Mg^{2+}	(ion2)E2:P, ATP(m)	3.46	2.31
3FPB [16]	K^+, ATP, MgF_4^{2-}, Mg^{2+}, CPA	(ion2)E2:P, ATP(m)	3.44	2.25
2C88 [17]	Tg, Mg^{2+}, Na^+, AMPPCP	E2	5.20	3.94

Tg = Thapsigargin, CPA = Cyclopiazonic acid

RMSD values were determined via structural comparison of Cα molecules of KdpB N, P and A domains and SERCA N, P and A domains in COOT[18]. The values presented in the above table range from 2.95 – 3.91 (1T5T & 4H1W) for state 1 compared to SERCA E1 structures and from 2.07 – 3.94 (1XP5 & 2C88) for state 2 compared to SERCA E2 structures. In each individual case SERCA E1 structures agreed better with state 1, while SERCA E2 structures agreed best with state 2. The overall rather high RMSDs are a result of already rather poor fits of the individual N, P, and A domains of state 1 and state 2 superimposed to E1 and E2 structures of SERCA, reflecting the structural differences of both proteins. The best scores of the individual domains were 3.72 for the N domain, 1.22 for the P domain, and 3.10 for the A domain, achieved for state 1 in comparison to 1T5T. In state 2 best scores were achieved in comparison to 1XP5 with 1.72 for the N domain, 1.07 for the P domain, and 1.70 for the A domain, respectively.

Supplementary Table 3 Mass spectrometry results for the analysis of Ser162 phosphorylation in KdpB.

		Replicate 1			Replicate 2		
**Site**	**Sequence**	**Total PSMs**	**# PSMs**	**phosphoRS probability**	**Total PSMs**	**# PSMs**	**phosphoRS probability**
**Phosphory-lated**	KGDIVLVEAGDIIPcDGEVIEGGASVDESAITGE**s**APVIR	**11**	10	96.9	**63**	47	99.9
	GDIVLVEAGDIIPcDGEVIEGGASVDESAITGE**s**APVIR		1	97		16	99.9
**Non-Phosphory-lated**	KGDIVLVEAGDIIPcDGEVIEGGASVDESAITGESAPVIR	**2**	2	N/A	**7**	5	N/A
	GDIVLVEAGDIIPcDGEVIEGGASVDESAITGESAPVIR		0	N/A		2	N/A

Sequence: amino acid sequence for the identified peptide. *PSMs*: spectral counts (total number of spectra matching to that peptide in a particular sample). *phosphoRS probability*: Phopshorylation assignment probability for Ser162 as calculated by PhosphoRS program.

Phosphorylation of KdpB at position Ser162 was confirmed for two biological replicates. Mascot searches were performed considering Ser, Thr, Tyr and Asp as putative phosphorylation sites. PhosphoRS was used for confident phosphorylation site localization[19]. Two different peptides; with and without a missed Trypsin cleavage site (Lys128); undoubtedly mapping to Ser162 were found to be phosphorylated. In addition, PhosphoRS assigned the phosphorylation to Ser162 in those peptides with a very high confidence score (>95%). Peptides carrying non-phosphorylated Ser162 were also detected but spectral counting data suggests that the phosphorylated form is much more abundant than the non-phosphorylated (*PSMs* parameter). Differences in the ionization and fragmentation of peptides may also account for this difference, and therefore caution should be taken when considering quantitative differences with such data[20].

Supplementary Table 4 ATPase assay with different inhibitor combinations.

Condition: SEC buffer, 2 mM Mg^{2+}-ATP +	µmol P_i mg^{-1} min^{-1}	% residual activity
1 mM KCl	1.28±0.04	100.0
-	0.47±0.03	36.5
1 mM KCl, 1 mM AMPPCP	1.12±0.04	87.1
1 mM KCl, 5 mM AlF_4^-	-0.01±0.02	-0.8
1 mM KCl, 1 mM AMPPCP, 5 mM AlF_4^-	0.00±0.03	-0.1

ATPase assays were performed directly after protein purification. 0.25 µg of protein was pre-incubated in the listed conditions for 5 min at 4°C. Reactions were started by the addition of 2 mM Mg^{2+}-ATP. Presented values are averages of three background-corrected (w/o KdpFABC) technical replicates ± SD. Protein activity in the presence of 1 mM KCl and 2 mM Mg^{2+}-ATP was set to 100%. Residual activity (36.5%) in the absence of KCl is caused by potassium contaminations in the nanomolar range since the K_M for K^+ of KdpFABC is ≤ 2µM. While AlF_4^- plus AMPPCP and AlF_4^-, respectively, inhibited ATP hydrolysis completely, treatment with AMPPCP alone led to a residual activity of 87.1% indicating a competition with ATP.

Supplementary References:

1 Scheres, S. H. RELION: implementation of a Bayesian approach to cryo-EM structure determination. *Journal of structural biology* **180**, 519-530, doi:10.1016/j.jsb.2012.09.006 (2012).

2 Kimanius, D., Forsberg, B. O., Scheres, S. H. & Lindahl, E. Accelerated cryo-EM structure determination with parallelisation using GPUs in RELION-2. *eLife* **5**, doi:10.7554/eLife.18722 (2016).

3 Cao, Y. *et al.* Crystal structure of a potassium ion transporter, TrkH. *Nature* **471**, 336-340, doi:10.1038/nature09731 (2011).

4 Ho, B. K. & Gruswitz, F. HOLLOW: generating accurate representations of channel and interior surfaces in molecular structures. *BMC structural biology* **8**, 49, doi:10.1186/1472-6807-8-49 (2008).

5 Smart, O. S., Neduvelil, J. G., Wang, X., Wallace, B. A. & Sansom, M. S. HOLE: a program for the analysis of the pore dimensions of ion channel structural models. *Journal of molecular graphics* **14**, 354-360, 376 (1996).

6 Kozlikova, B. *et al.* CAVER Analyst 1.0: graphic tool for interactive visualization and analysis of tunnels and channels in protein structures. *Bioinformatics* **30**, 2684-2685, doi:10.1093/bioinformatics/btu364 (2014).

7 Nyblom, M. *et al.* Crystal structure of Na^+, K^+-ATPase in the Na^+-bound state. *Science* **342**, 123-127, doi:10.1126/science.1243352 (2013).

8 Abe, K. *et al.* The cryo-EM structure of gastric H^+,K^+-ATPase with bound BYK99, a high-affinity member of K^+-competitive, imidazo[1,2-a]pyridine inhibitors. *Scientific reports* **7**, 6632, doi:10.1038/s41598-017-06698-8 (2017).

9 Winther, A. M. *et al.* The sarcolipin-bound calcium pump stabilizes calcium sites exposed to the cytoplasm. *Nature* **495**, 265-269, doi:10.1038/nature11900 (2013).

10 Sorensen, T. L., Moller, J. V. & Nissen, P. Phosphoryl transfer and calcium ion occlusion in the calcium pump. *Science* **304**, 1672-1675, doi:10.1126/science.1099366 (2004).

11 Toyoshima, C. & Mizutani, T. Crystal structure of the calcium pump with a bound ATP analogue. *Nature* **430**, 529-535, doi:10.1038/nature02680 (2004).

12 Olesen, C. *et al.* The structural basis of calcium transport by the calcium pump. *Nature* **450**, 1036-1042, doi:10.1038/nature06418 (2007).

13 Toyoshima, C., Norimatsu, Y., Iwasawa, S., Tsuda, T. & Ogawa, H. How processing of aspartylphosphate is coupled to lumenal gating of the ion pathway in the calcium pump. *Proceedings of the National Academy of Sciences of the United States of America* **104**, 19831-19836, doi:10.1073/pnas.0709978104 (2007).

14 Olesen, C., Sorensen, T. L., Nielsen, R. C., Moller, J. V. & Nissen, P. Dephosphorylation of the calcium pump coupled to counterion occlusion. *Science* **306**, 2251-2255, doi:10.1126/science.1106289 (2004).

15 Toyoshima, C., Nomura, H. & Tsuda, T. Lumenal gating mechanism revealed in calcium pump crystal structures with phosphate analogues. *Nature* **432**, 361-368, doi:10.1038/nature02981 (2004).

16 Laursen, M. *et al.* Cyclopiazonic acid is complexed to a divalent metal ion when bound to the sarcoplasmic reticulum Ca^{2+}-ATPase. *J Biol Chem* **284**, 13513-13518, doi:10.1074/jbc.C900031200 (2009).

17 Jensen, A. M., Sorensen, T. L., Olesen, C., Moller, J. V. & Nissen, P. Modulatory and catalytic modes of ATP binding by the calcium pump. *The EMBO journal* **25**, 2305-2314, doi:10.1038/sj.emboj.7601135 (2006).

18 Emsley, P. & Cowtan, K. Coot: model-building tools for molecular graphics. *Acta crystallographica. Section D, Biological crystallography* **60**, 2126-2132, doi:10.1107/S0907444904019158 (2004).

19 Taus, T. *et al.* Universal and confident phosphorylation site localization using phosphoRS. *Journal of proteome research* **10**, 5354-5362, doi:10.1021/pr200611n (2011).

20 Solari, F. A., Dell'Aica, M., Sickmann, A. & Zahedi, R. P. Why phosphoproteomics is still a challenge. *Molecular bioSystems* **11**, 1487-1493, doi:10.1039/c5mb00024f (2015).
