# Supplementary material for: Cryo-EM structures of KdpFABC suggest a K+ transport mechanism via two inter-subunit half-channels
Source: Nat Commun. 2018 Nov 26;9:4971. doi: 10.1038/s41467-018-07319-2 (PMC6255902; doi:10.1038/s41467-018-07319-2)
Supplement: Supplementary file 3 — Description of Additional Supplementary Files [file 41467_2018_7319_MOESM3_ESM.docx]

**Title:** Supplementary Movie 1
**Description:** Conformational changes during transport in KdpFABC. Morph between the nucleotide-free E1 state [5MRW], the late nucleotide-bound E1 state (state 1) and the late E2-P state (state 2) of the KdpFABC complex. Color code is as depicted in Fig 1; potassium ions dark purple spheres; entrance and exit tunnels as pink densities. Respective structural features and relevant residues are indicated when applicable.
